# A Predictive Model of Live Birth Based on Obesity and Metabolic Parameters in Patients With PCOS Undergoing Frozen-Thawed Embryo Transfer

**DOI:** 10.3389/fendo.2021.799871

**Published:** 2022-01-12

**Authors:** Xiaohua Jiang, Ruijun Liu, Ting Liao, Ye He, Caihua Li, Peipei Guo, Ping Zhou, Yunxia Cao, Zhaolian Wei

**Affiliations:** ^1^ Department of Obstetrics and Gynecology, The First Affiliated Hospital of Anhui Medical University, Hefei, China; ^2^ National Health Commission (NHC) Key Laboratory of Study on Abnormal Gametes and Reproductive Tract, Anhui Medical University, Hefei, China; ^3^ Anhui Province Key Laboratory of Reproductive Health and Genetics, Anhui Medical University, Hefei, China

**Keywords:** polycystic ovary syndrome, obesity, nomogram, total cholesterol, triglycerides, insulin resistance

## Abstract

**Aims:**

To determine the clinical predictors of live birth in women with polycystic ovary syndrome (PCOS) undergoing frozen-thawed embryo transfer (F-ET), and to determine whether these parameters can be used to develop a clinical nomogram model capable of predicting live birth outcomes for these women.

**Methods:**

In total, 1158 PCOS patients that were clinically pregnant following F-ET treatment were retrospectively enrolled in this study and randomly divided into the training cohort (n = 928) and the validation cohort (n = 230) at an 8:2 ratio. Relevant risk factors were selected *via* a logistic regression analysis approach based on the data from patients in the training cohort, and odds ratios (ORs) were calculated. A nomogram was constructed based on relevant risk factors, and its performance was assessed based on its calibration and discriminative ability.

**Results:**

In total, 20 variables were analyzed in the present study, of which five were found to be independently associated with the odds of live birth in univariate and multivariate logistic regression analyses, including advanced age, obesity, total cholesterol (TC), triglycerides (TG), and insulin resistance (IR). Having advanced age (OR:0.499, 95% confidence interval [CI]: 0.257 – 967), being obese (OR:0.506, 95% CI: 0.306 - 0.837), having higher TC levels (OR: 0.528, 95% CI: 0.423 - 0.660), having higher TG levels (OR: 0.585, 95% CI: 0.465 - 737), and exhibiting IR (OR:0.611, 95% CI: 0.416 - 0.896) were all independently associated with a reduced chance of achieving a live birth. A predictive nomogram incorporating these five variables was found to be well-calibrated and to exhibit good discriminatory capabilities, with an area under the curve (AUC) for the training group of 0.750 (95% CI, 0.709 - 0.788). In the independent validation cohort, this model also exhibited satisfactory goodness-of-fit and discriminative capabilities, with an AUC of 0.708 (95% CI, 0.615 - 0.781).

**Conclusions:**

The nomogram developed in this study may be of value as a tool for predicting the odds of live birth for PCOS patients undergoing F-ET, and has the potential to improve the efficiency of pre-transfer management.

## Introduction

Polycystic ovary syndrome (PCOS) is among the most prevalent endocrine disorders impacting women of reproductive age, resulting in symptoms that include hyperandrogenism, oligoovulation, or anovulation. PCOS rates vary substantially among different populations with an estimated PCOS incidence of 5.6% in China at present (1).

Roughly 80% of women who suffer from anovulatory infertility are diagnosed with PCOS (2). Owing to irregular menstruation, PCOS patients are more likely to experience infertility and to utilize assisted reproductive technology (ART) for conception as compared to individuals without PCOS (3). Even after conception, PCOS patients are at a higher risk of adverse pregnancy-related outcomes such as miscarriage, gestational diabetes, pre-eclampsia, and preterm delivery (3–6). Potential causes for these adverse outcomes have been suggested to include IR, obesity, hyperandrogenism, dyslipidemia, and chronic low-grade inflammation (4). Metabolic abnormalities which impact PCOS patient ART outcomes can result in multiple failed embryo transfers, which can be financially, physically, and emotionally taxing for the affected family. It is thus essential that individual risk factors be managed prior to embryo transfer in PCOS patients in order to reduce the odds of negative pregnancy outcomes.

In order to determine which PCOS patients are at higher risk of adverse pregnancy outcomes and to guide pre-transfer treatment efforts, it is critical that a reliable predictive model incorporating metabolic and clinical variables associated with the live birth rate among PCOS patients be established. Predicting the odds of live birth following frozen-thawed embryo transfer (F-ET) may be of particular value as it would enable clinicians to more optimally manage infertility associated with PCOS. Accurately gauging individual patient risk may also be able to alleviate some of the anxiety associated with the unpredictability of pregnancy outcomes. A nomogram would enable highly accurate risk estimation in an evidence-based and individualized manner, making the development of such a tool ideal in this clinical context.

Nomograms are easy-to-use tools that can be readily employed to guide patient management and associated decision-making. This study is the first to our knowledge to have developed a nomogram aimed at individually estimating the live birth rates for PCOS patients undergoing F-ET based on an analysis of the relationship between key metabolic and clinical risk factors and live birth rates among these patients.

## Materials and Methods

### Patient Population

Between January 2018 and September 2020, all women with PCOS who became clinically pregnant following F-ET performed at the First Affiliated Hospital of Anhui Medical University (Anhui, China) were retrospectively included in this study. Patients were diagnosed with PCOS in accordance with the 2003 Rotterdam criteria based on the presence of a minimum of two of the following: polycystic ovaries, biochemical or clinical evidence of hyperandrogenism, and oligo-/anovulation (7). All patients underwent testing for testosterone levels. Some of the patients’ androstenedione, DHEAS and SHBG were tested and FAI was calculated when data was available. All of these indicators were used to assess biochemical hyperandrogenism. Clinical hyperandrogenism was assessed based on the presence of hirsutism, acne, and androgen-related alopecia. Patients were excluded from this study if they exhibited reproductive system malformations, endometriosis, endocrine disorders (including clinically diagnosed hypertension, Cushing syndrome, hyperprolactinemia, thyroid dysfunction, and congenital adrenal hyperplasia), chromosomal diseases, or other factors that could potentially affect pregnancy outcomes. In addition, women with male partners diagnosed with severe male factor infertility, non-obstructive azoospermia, or genetic disorders which may be transmitted by spermatozoa were excluded. Based on these criteria, 1158 patients were included in this study. The complete dataset for the included patients was randomly partitioned into a training cohort and a validation cohort using R ‘set. seed ()’ command. Eighty percent of patients (n = 928) were grouped into a training cohort used for live birth rate prediction and 20% (n = 230) were grouped into a validation cohort used for internal model validation. Written informed consent was provided by each couple for F-ET, and the study was approved by the hospital Ethics Committee (reference: Quick-PJ 2021-10-14).

### Biochemical Analysis

The hexokinase method and electrochemiluminescence were respectively used to measure levels of fasting plasma glucose (FPG) and fasting insulin (FIN). A turbidimetric inhibition immunoassay approach was employed for measurements of total cholesterol (TC), high-density lipoprotein cholesterol (HDL-C), and triglycerides (TG). Westergren’s international standard method was employed to calculate the erythrocyte sedimentation rate (ESR) for each patient. The fasting measurement of HDL-C, TC, TG, plasma glucose, and insulin were measured within three months prior to embryo transfer.

Chemiluminescence assays were employed to measure basal testosterone (T), follicle-stimulating hormone (FSH), estradiol (E_2_), and luteinizing hormone (LH) levels on day 2-3 of the menstrual cycle.

### Controlled Ovarian Hyperstimulation and F-ET Procedure

All the included patients received routine ovarian stimulation, oocyte retrieval, and fertilization. Patients were stimulated using a long GnRH agonist protocol or an antagonist protocol. Follicle growth was regularly monitored *via* transvaginal ultrasound, and the levels of E_2_, LH, and progesterone were monitored. When at least two leading follicles were ≥ 18 mm, 5000–10000 IU human chorionic gonadotropin was administered. Oocytes were aspirated 36 hours later and embryos were cryopreserved on day 5 or 6.

Natural or artificial F-ET cycles were conducted for patients based upon their individual fertility situation. Gardner blastocysts score was used to evaluate the quality of the embryo, ≥ 3BB was considered as good-quality embryo as previously described (8).

For natural F-ET cycles, ovulation was monitored from day 8-12 of the menstrual cycle *via* routine transvaginal ultrasound, with monitoring frequency being based upon a combination of follicle sizes, urinary LH levels, serum LH levels, and E_2_ levels until ovulation. Human chorionic gonadotropin was administered to induce ovulation if appropriate. On the day of ovulation, progesterone (40-80 mg/d) was administered at a dose appropriate to the weight of the individual patient in order to prevent luteal phase defects. On day 5 post-ovulation, at least one good-quality embryo was thawed and transferred.

For artificial F-ET cycles, patients were administered estradiol valerate (3-4 mg/d) beginning on the third day of menstruation to prepare the endometrium, which was monitored beginning on day 8 of the menstrual cycle *via* routine transvaginal ultrasound. When unsatisfactory endometrial growth was observed, the dose of estrogen was increased accordingly. When the endometrium reached a thickness of 8 mm, progesterone (40-80 mg/d) was administered, and at least one good-quality embryo was thawed and transferred on day 5 following progesterone administration.

Serum human chorionic gonadotropin levels were measured two weeks after embryo transfer and clinical pregnancy was confirmed by ultrasound 30 days after embryo transfer. If patients were pregnant, luteal support medication was gradually stopped 10 weeks after pregnancy. If they were not, luteal support was stopped immediately.

### Outcome Measures

IR was diagnosed in these PCOS patients based upon homeostatic model assessment of insulin resistance (HOMA-IR) values, which were calculated as follows: FPG × FIN/22.5. IR was diagnosed based on a HOMA-IR value > 2.69, in accordance with the findings of a large community-based analysis of PCOS patients conducted in China (1). Transvaginal ultrasound imaging was used to confirm clinical pregnancy and heartbeat 30 days after F-ET. Live birth was defined by the viable delivery of at least one newborn.

### Statistical Analysis

SPSS 23.0 and R (v 4.0.1) were employed for all statistical analyses. Student’s t-tests, Mann-Whitney U tests and Chi-squared tests were used to compare the patient characteristics between training cohort and validation cohort as appropriate.

### Model Development

The live birth rates of PCOS patients following F-ET were the primary outcome for this study. The nomogram was developed using data from the training cohort (928 patients). First, we applied the univariate analyses to select the variables with the greatest predictive value as predictors of live birth from the included baseline characteristics. Then, those variables with a *P*-value < 0.05 in univariate analyses were fitted into a backward stepwise multivariable logistic regression (MLR) model to re-evaluate the impact of these factors on the live birth of PCOS patients. *P* values in this multivariable analysis were based on the Likelihood test. A *P*-value < 0.05 was considered significant. This MLR approach was used to compute coefficient values for each of the independent predictors, and a final nomogram was then developed using the R platform as a graphical representation of this predictive MLR model.

### Model Evaluation

Nomogram performance was quantified based upon the results of calibration and discrimination analyses. The area under the receiver operating characteristic (ROC) curve (AUC) values and corresponding 95% confidence intervals (CIs) were determined to gauge the predictive accuracy of this nomogram. Calibration was assessed using calibration curves, which graphically represented the association between actual and predictive probabilities. The utility and accuracy of this nomogram were confirmed using data from the validation patient cohort (n = 230). ROC curves and the calibration curves were developed using the R platform.

## Results

### Patient Characteristics

In total, 1158 PCOS patients meeting the study inclusion criteria were enrolled in this analysis, including 928 and 230 patients in the training and validation cohorts, respectively. Patient characteristics and clinical outcomes are detailed in **Table 1**. There were no significant differences between these two cohorts, with live births being recorded in 744 (80.17%) and 182 (79.13%) patients in the training and validation cohorts, respectively.

**Table 1:** Participant characteristics.

	Training cohort (n = 928)	Validation cohort (n = 230)	*P* value
Age, years	28.84 ± 3.45	28.81 ± 3.52	0.813
Advanced Age			0.419
≥ 35	48 (5.7)	15 (6.52)	
< 35	880 (94.83)	215 (93.48)	
Duration of infertility, years	3.39 ± 2.31	3.39 ± 2.30	0.918
Type of infertility, n (%)			0.887
primary infertility	548 (59.05)	137 (59.57)	
secondary infertility	380 (40.95)	93 (40.43)	
Reproductive History			
pregnancy, n (%)			0.887
0	548 (59.05)	137 (59.57)	
≥ 1	380 (40.95)	93 (40.43)	
miscarriage, n (%)			0.688
0	670 (72.20)	163 (70.87)	
≥ 1	258 (27.80)	67 (29.13)	
ectopic pregnancy, n (%)			0.213
0	839 (90.41)	214 (93.04)	
≥ 1	89 (9.59)	16 (6.96)	
BMI, Kg/m^2^	23.62 ± 3.28	23.40 ± 3.22	0.364
Obesity, n (%)			0.322
yes	97 (10.45)	19 (8.26)	
No	831 (89.55)	211 (91.74)	
ESR (mm/h)	10.90 ± 6.79	10.74 ± 6.11	0.842
FSH, IU/L	6.38 ± 1.70	6.46 ± 1.66	0.528
LH, IU/L	9.31 ± 5.82	9.18 ± 5.60	0.921
LH/FSH	1.53 ± 0.98	1.46 ± 0.93	0.255
E_2,_ pmol/L	167.66 ± 107.52	176.96 ± 110.48	0.178
T, nmol/L	1.92 ± 0.86	1.96 ± 0.89	0.554
TC, mmol/L	4.51 ± 0.86	4.47 ± 0.80	0.439
TG, mmol/L	1.44 ± 0.84	1.49 ± 0.98	0.762
HDL-C, mmol/L	1.30 ± 0.37	1.33 ± 0.41	0.330
FPG, mmol/L	5.37 ± 0.59	5.36 ± 0.65	0.581
FIN, mU/L	12.92 ± 7.19	12.58 ± 6.27	0.767
HOMA-IR	3.12 ± 1.83	3.04 ± 1.65	0.751
IR, n (%)			0.659
yes	471 (50.75)	113 (49.13)	
no	457 (49.25)	117 (50.87)	
Number of transferred embryos, n (%)			0.654
1	443 (47.74)	106 (46.09)	
> 1	485 (52.26)	124 (53.91)	
Type of circle, n (%)			0.975
natural F-ET cycles	57 (6.14)	14 (6.09)	
artificial F-ET cycles	871 (93.86)	216 (93.91)	
Endometrium thickness, mm	10.29 ± 1.40	10.33 ± 1.24	0.119
Live birth, n (%)			0.724
yes	744 (80.17)	182 (79.13)	
No	184 (19.83)	48 (20.87)	

Continuous variables are expressed as mean ± standard deviation, categorical variables as absolute frequencies, n (%). BMI, body mass index; E_2_, estradiol; ESR, erythrocyte sedimentation rate; F-ET, frozen-thawed embryo transfer; FIN, fasting insulin; FPG, fasting plasma glucose; FSH, follicle-stimulating hormone; HDL-C, high density lipoprotein cholesterol; HOMA-IR, homeostatic model assessment of insulin resistance; LH, luteinizing hormone; T, testosterone; TC, total cholesterol; TG, triglycerides.

### Logistic Regression Analyses Reveal Five Physiological Parameters Correlated With Live Birth Rates

**Figure 1:**
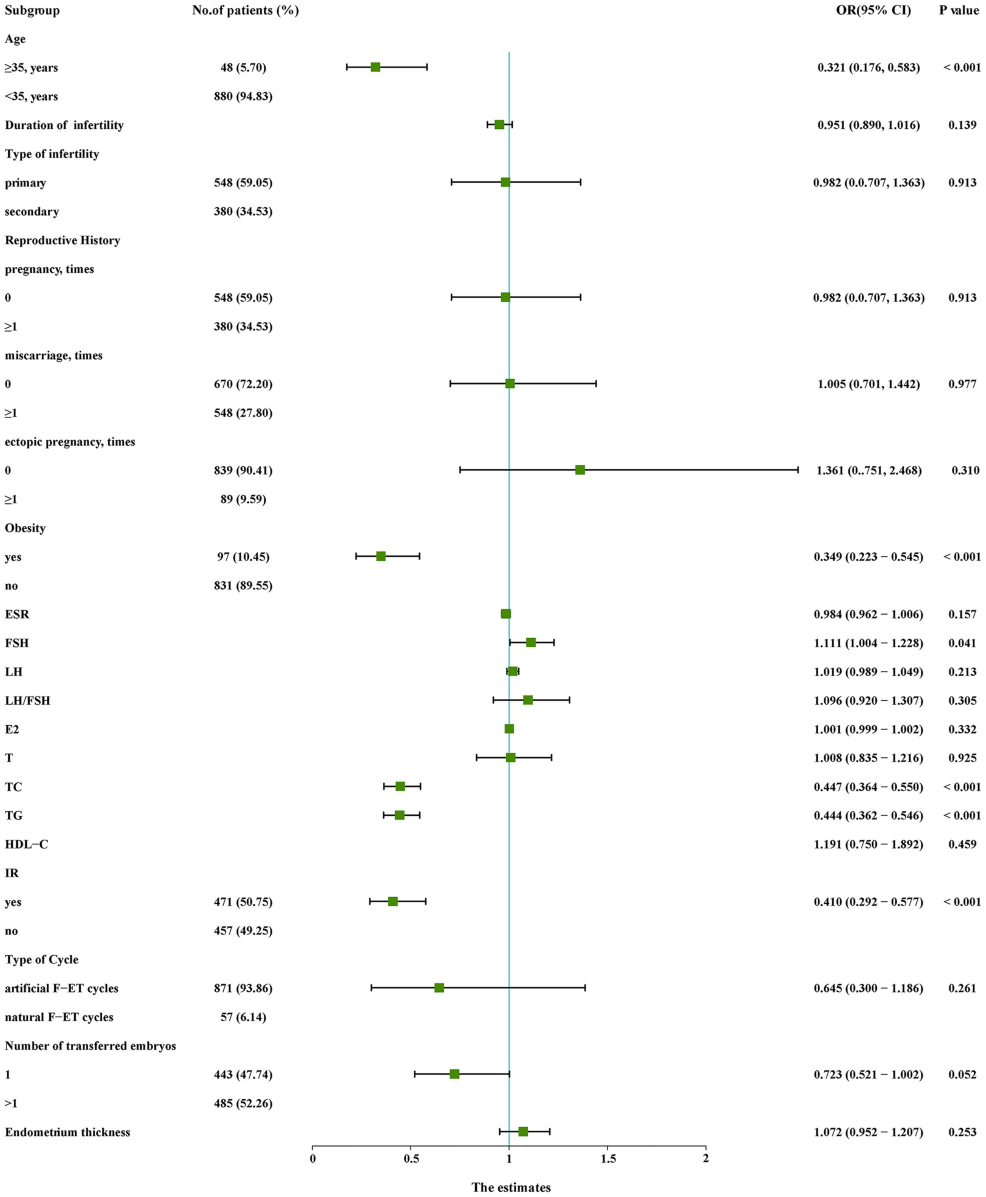
Univariate logistic regression analyses of relevant variables in the training cohort. CI, confidence interval; E_2_, estradiol; ESR, erythrocyte sedimentation rate; F-ET, frozen-thawed embryo transfer; FSH, follicle-stimulating hormone; HDL-C, high-density lipoprotein cholesterol; IR, insulin resistance; LH, luteinizing hormone; OR, odds ratio; T, testosterone; TC, total cholesterol; TG, triglycerides.

Initial univariate analyses (**Figure 1**) indicated that advanced age, obesity, higher TC levels, higher TG levels, and IR were associated with lower odds of live birth (*P* < 0.05), whereas higher basal FSH levels were associated with increased odds of live birth (*P* < 0.05). A subsequent multivariate analysis revealed that advanced age (OR: 0.499, 95% CI: 0.257 – 0.967), obesity (OR: 0.506, 95% CI: 0.306 - 0.837), TC (OR: 0.528, 95% CI: 0.423 - 0.660), TG (OR: 0.585, 95% CI: 0.465 – 0.737), and IR (OR: 0.611, 95% CI: 0.416 - 0.896) were independently related to the odds of live birth for PCOS patients undergoing F-ET (**Table 2**). Specifically, live birth rates were lower for women who were obese, IR, had advanced age, had higher TC levels, and had higher TG levels.

**Table 2:** Multivariate logistic regression analysis of live birth based on data in the training cohort.

	OR (95% CI)	*P* Value
Age		
≥35 vs <35	0.499 (0.257 - 0.967)	0.04
Obesity		
obesity vs non-obesity	0.506 (0.306 - 0.837)	0.008
TC	0.528 (0.423 - 0.660)	<0.001
TG	0.585 (0.465 - 0.737)	<0.001
IR		
IR vs non-IR	0.611 (0.416 - 0.896)	0.012
FSH		0.491

CI, confidence interval; FSH, follicle-stimulating hormone; IR, insulin resistance; OR, odd ratios; TC, total cholesterol; TG, triglycerides.

### Nomogram Development

**Figure 2:**
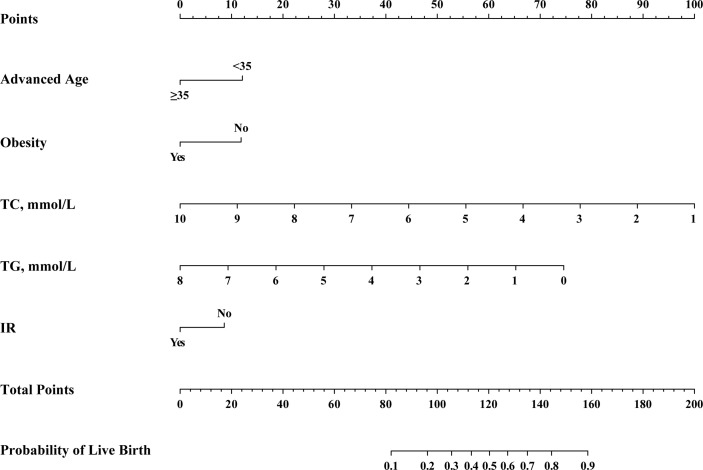
Nomogram for the pre-transfer prediction of live birth odds in PCOS patients undergoing F-ET. F-ET, frozen-thawed embryo transfer; FSH, follicle-stimulating hormone; IR, insulin resistance; TC, total cholesterol; TG, triglycerides.

The results of the above multivariate analysis were next used to construct a nomogram capable of predicting live birth rates (**Figure 2**). To use this nomogram, a vertical line was drawn from the appropriate point for each predictive factor up to the ‘Points’ scale. Point values for each variable were then summed together, and the total point value was used to determine the probability of live birth for a given patient. This nomogram provides patients with an easy-to-understand and comprehensive overview of the overall likelihood of achieving a live birth and the influence of individual factors on this outcome.

### Model Evaluation

**Figure 3:**
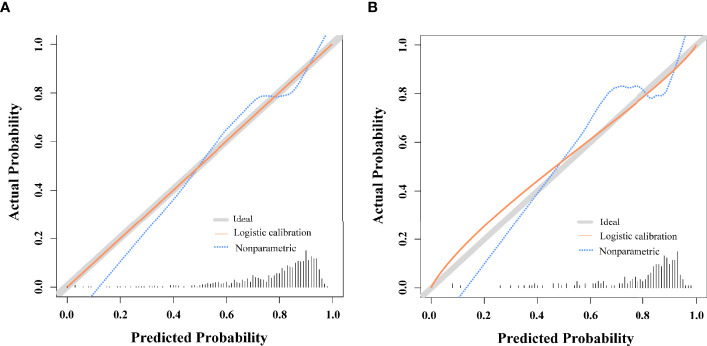
Nomogram Calibration. **(A)** Calibration curves examining the relationship between the predicted odds of live birth and actual live birth rates in the training cohort. **(B)** Calibration curves examining the relationship between the predicted odds of live birth and actual live birth rates in the validation cohort.

**Figure 4:**
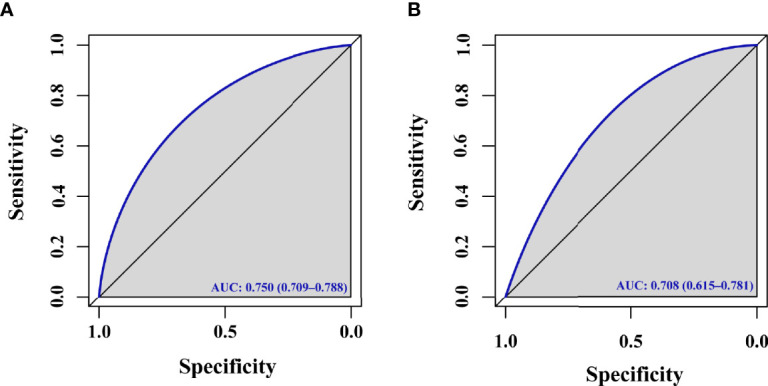
ROC curve. **(A)** ROC curve evaluating the ability of this model to predict live births for PCOS patients in the training cohort undergoing F-ET. **(B)** ROC curve evaluating the ability of this model to predict live births for PCOS patients in the validation cohort undergoing F-ET.

For the predictive model, calibration curves exhibited good consistency between predicted and actual results, consistent with appropriate calibration (**Figure 3A**). ROC curves exhibited an AUC of 0.750 (95% CI, 0.709 - 0.788) in the training cohort, consistent with the good discriminatory capabilities (**Figure 4A**). Calibration and ROC curves for this nomogram when used to analyze the validation cohort are shown in **Figures 3B**, **4B**. In this validation cohort, the model exhibited fair predictive performance with an AUC of 0.708 (95% CI, 0.615 - 0.781) (**Figure 4B**), consistent with fair discriminatory capabilities.

## Discussion

The nomogram developed herein is the first to our knowledge to have predicted live birth rates for PCOS patients following F-ET. This novel clinical model was developed based on the evaluation of clinical and metabolic parameters derived from 1158 PCOS patients, of whom 928 and 230 were respectively assigned to training and validation cohorts. The calibration and discriminatory capabilities of this nomogram were deemed satisfactory, and the final nomogram was prepared in the form of a user-friendly visual scale (**Figure 2**). This predictive model has several advantages, including the fact that it draws its predictions from analyses of clinical and laboratory data that are readily available and the fact that it incorporates key metabolic variables (TC, TG, and IR) which correspond to dynamic changes in the context of ART.

Our final predictive nomogram incorporated five variables (advanced age, obesity, serum TC, TG, and IR) as independent predictors of live birth rates in these patients, and these results were in line with those from other previously published studies. Advanced age has long been considered to be an important influential factor in the context of reproductive medicine. A prospective study determined that age is an independent predictor of live birth in women undergoing IVF treatment (9). Advanced age is related to many adverse factors that can affect oocyte quality, including cytoskeletal abnormalities, decrease numbers of mitochondria, abnormal spindle formation, aneuploidy, and zona pellucida dysfunction (10, 11). Minasi et al. emphasized that rates of aneuploidy rise by 10% every year with increasing maternal age, suggesting that this may be the primary cause of IVF cycle implantation failure and abortion (12).

Obesity has previously been linked to adverse pregnancy outcomes following ART treatment (13–16). Roughly half of all PCOS patients are overweight or obese (17), and a higher BMI is negatively correlated with live birth rates for PCOS patients undergoing ART treatment (18, 19). Leary et al. (20) determined that high maternal BMI was also associated with phenotypic changes in the embryo during the preimplantation period, with metabolic abnormalities more often being evident for embryos in women who were overweight or obese. Obesity may contribute to the incidence of negative pregnancy outcomes through the impairment of decidualization owing to defective autophagy, resulting in implantation abnormalities (21). Obesity can also reduce the number of M1 macrophages within the decidua parietalis, potentially contributing to the development of a proinflammatory microenvironment that may contribute to pregnancy failure (22). Weight loss has been conclusively shown to improve ART outcomes including rates of live birth (23).

We found that certain metabolic parameters were reliably associated with live birth rates for PCOS patients undergoing F-ET. Metabolic syndrome has been shown to negatively affect live birth rates for PCOS patients undergoing ART (24). PCOS patients commonly exhibit aberrant lipid profiles, including increased TC, TG, and LDL-C levels together with reductions in HDL-C levels (25, 26). Serum lipids have the potential to impact embryo quality and thereby shape ART outcomes, as evidenced by negative correlations between embryo quality and both TG and TC levels (27). In one recent analysis, higher TC levels were found to independently predict rates of live birth for patients with PCOS undergoing IVF/ICSI treatment (28). Higher serum TG levels before conception are also linked to lower live birth rates following IVF as compared to those women with normal TG levels (29). IR is another metabolism-associated factor that is common in PCOS patients and that was associated with live birth rates in our study population. IR has been independently linked to live birth odds among PCOS patients (30). In prior reports, a combination of IR and hyperandrogenism was shown to cause placental and uterine dysfunction *via* by inducing ferroptotic cell death (31). In addition to causing physical damage, IR can also negatively impact the mental health of PCOS patients. For example, Greenwood et al. were able to identify an independent link between IR and the incidence of depression among individuals with PCOS (32). Managing abnormal metabolic parameters through diet, exercise, behavioral changes, and pharmacological treatment can lead to better pregnancy outcomes in addition to decreasing the risk of developing metabolic disorders (33, 34).

While a prior study established a model designed to predict the live birth rates of PCOS patients undergoing both fresh embryo transfer and F-ET based on overweight and lipid metabolism-related parameters (28), no specific metabolic parameter-related models have been developed to predict the odds of live birth for PCOS patients following F-ET. In addition, we incorporated glucose metabolism related parameters in the present study in an effort to strengthen our developed model. A reliable nomogram would be of clear value as a tool for use when determining whether a patient should transfer an embryo during a given cycle or should cancel their cycle and attempt to improve relevant metabolism-related metrics prior to embryo transfer. The nomogram developed herein is a novel tool that has been shown to reliably predict live birth rates for PCOS patients following F-ET. This tool may also be able to aid clinicians in maximizing available data to appropriately treat PCOS patients who are less likely to achieve a live birth following F-ET by postponing or canceling cycles for these individuals if appropriate.

This study has several strengths that make our conclusions more robust. For one, our sample size was relatively large. In addition, this study is the first to our knowledge to have established a model for individually evaluating the odds of live birth for PCOS patients following F-ET treatment. Third, a wide range of potential risk factors was considered when constructing this model. Despite these strengths, there are nonetheless certain limitations to our analyses. First, this was a single-center study, and validation using datasets from other centers will thus be necessary in the future. Second, this was a retrospective study and it is thus potentially susceptible to recall bias, such that a large-scale prospective study will be essential to confirm the reliability of the nomogram developed herein.

In summary, we herein established an accurate and objective model capable of predicting live birth rates for PCOS patients undergoing F-ET. Following the external validation of this nomogram, it may be of value as a tool for guiding patient management and ART strategy selection in the clinic.

## Data Availability Statement

The raw data supporting the conclusions of this article will be made available by the authors, without undue reservation.

## Ethics Statement

The studies involving human participants were reviewed and approved by First Affiliated Hospital of Anhui Medical University. The patients/participants provided their written informed consent to participate in this study.

## Author Contributions

ZW designed the study. XJ, TL, and RL carried out the data collection and statistical analysis. XJ wrote the paper. TL, PG, and CL helped with the data collection. PZ, YH, and YC contributed to the reviewing of the paper. All authors contributed to the article and approved the submitted version.

## Funding

This work was supported by the University Natural Science Research Project of Anhui Province (KJ2020A0201).

## Conflict of Interest

The authors declare that the research was conducted in the absence of any commercial or financial relationships that could be construed as a potential conflict of interest.

## Publisher’s Note

All claims expressed in this article are solely those of the authors and do not necessarily represent those of their affiliated organizations, or those of the publisher, the editors and the reviewers. Any product that may be evaluated in this article, or claim that may be made by its manufacturer, is not guaranteed or endorsed by the publisher.
